# Reprogramming microglia in sepsis-associated encephalopathy: from pathological dysfunction to therapeutic restoration

**DOI:** 10.3389/fimmu.2026.1804207

**Published:** 2026-04-16

**Authors:** Chen He, Hui Shi, Zhijie Yu, Zhiqiang Jiao, Jin Li, Fei Yang

**Affiliations:** 1 Chifeng Clinical Medical College of Inner Mongolia Medical University, Chifeng, China; 2 Department of Critical Care Medicine, Chifeng Municipal Hospital, Chifeng, China; 3 Department of Emergency Medicine, Chifeng Municipal Hospital, Chifeng, China

**Keywords:** microglia, sepsis-associated encephalopathy, cellular reprogramming, neuroinflammation, epigenetic regulation, inflammatory response, immune surveillance

## Abstract

Microglia, being the resident immunological sentinels of the central nervous system (CNS), play a critical role in both the pathogenesis and progression of CNS health and disease. In sepsis-associated encephalopathy (SAE), it is increasingly evident that the phenotype and function of microglia may change from a neuroprotective phenotype to a potential effector phenotype with neurotoxic potential. However, the exact mechanism by which this change is mediated remains to be understood. The current body of research is mostly focused on the hyperactivation of microglia, while the mechanism by which a pathological state develops from a systemic response remains to be understood. This limits the ability to design precise therapeutic strategies to target this cell population. In this regard, a framework of pathological state reprogramming is proposed to systematically evaluate potential mechanisms of microglial dysfunction in SAE. In this review, we will attempt to integrate the body of knowledge from single cell multi-omics, functional genetics, and *in vivo* imaging to determine the molecular characteristics of SAE-associated microglial states and potential functional alterations such as synaptic pruning. Further discuss the key factors that contribute to this evolution, such as inflammatory signaling, transcriptional and epigenetic regulation networks, and metabolic remodeling; and based on this basis, discuss multi-level therapeutic strategies for reversing the pathological state and restoring their protective role. These include potential strategies such as epigenetic and metabolic pathway modulation, CRISPR-mediated gene regulation, and cell therapies. These strategies consider microglia as a functional entity that has plasticity and can be modulated. The mechanistic basis developed in this review not only helps in understanding the pathology of SAE but also provides a basis for developing novel inflammatory modulation therapies for reversing the pathological state of microglia and restoring their protective surveillance role. This mechanistic basis for therapeutic innovation has a wide range of implications for developing intervention strategies for neuroinflammatory disorders.

## 1 Introduction

Sepsis-associated encephalopathy (SAE) is one of the most severe neurological complications of sepsis, with an incidence rate as high as 70% in intensive care units (ICU) (1). Clinical manifestations primarily include acute impaired consciousness, cognitive dysfunction, and delirium, which are closely associated with significantly increased mortality and long-term neurocognitive sequelae (2). However, specific therapeutic interventions for SAE remain lacking in clinical practice, with primary strategies still confined to systemic infection control and symptomatic support (3). This reflects critical gaps in our understanding of the precise neuropathological mechanisms underlying SAE.

Microglia are derived from yolk sac precursors and colonize the CNS during early embryonic development. They persist as the sole immune cells within the brain parenchyma throughout the entire lifespan (4, 5). Under physiological circumstances, microglia continually survey the microenvironment within the neural compartment via their highly dynamic structures, accurately controlling synaptic pruning while efficiently removing any debris (6, 7). Recent studies have revealed that microglia possess the fundamental ability to alter their phenotypic states in response to environmental cues, a property crucial for disease progression, repair, and development. In the context of neurodegenerative diseases, microglia have been observed to transform from their homeostatic sentinel phenotype into a disease-associated microglial (DAM) phenotype, a transition mediated by reprogramming of the transcriptional and epigenetic landscape (8). Studies employing two classical sepsis models-–lipopolysaccharide (LPS) injection and cecal ligation and puncture (CLP)-–have revealed a conserved DAM state that profoundly impacts the CNS immune phenotype.

However, in acute inflammation-driven SAE, whether microglia undergo similar systemic functional reprogramming, along with the dynamics of their state transitions and molecular regulatory mechanisms, remains to be elucidated. Current research suggests that the microglial response in SAE may transcend the traditional concept of activation, indicating the presence of more complex functional reprogramming (9).

Based on the above, this review article proposes the following theoretical framework for the pathological functional reprogramming of microglia in SAE: First, it will integrate the latest cutting-edge omics data to precisely define the molecular and functional characteristics of the reprogrammed state of microglia (10). Next, it will explore the underlying multi-level regulatory networks of the reprogramming of microglia (11). Finally, it will focus on the review of the latest innovative therapeutic strategies, especially those of the pioneering kind targeting the reversal of the reprogramming of microglia to restore, if not even enhance, the functional roles of the latter (9, 10). Such an approach not only helps deepen the understanding of the pathogenesis of SAE but also presents new insights for the development of precision medicine targeting the CNS immune microenvironment. Based on the systematic exploration of the inflammatory reprogramming of microglia in SAE, this paper hopes to provide an answer to the fundamental inflammatory biology question of how systemic inflammation, once it becomes acute, constantly reprograms the functions of the resident immune cells of the brain, laying the foundation for the development of related therapeutic strategies.

## 2 Characteristics of pathological reprogramming

### 2.1 Systemic reconstruction of molecular identity

The use of single-cell transcriptomics has transformed our knowledge of microglial activation in SAE. Investigations employing classical sepsis models, such as LPS challenge and CLP, have consistently identified a conserved DAM state (12). The most striking characteristic of this state is the profound reduction in genes that encode the purinergic receptor P2RY12, the transmembrane protein TMEM119, and the chemokine receptor CX3CR1 (13). These proteins work in concert to maintain the resting surveillance role of microglia and the neuron-glia interface; their reduced expression indicates that the cell is no longer in a state of homeostasis.

Concurrently, a distinct gene expression profile becomes synergistically activated. This profile not only includes classical inflammatory response elements but is also enriched for genes involved in lipid metabolism, extracellular matrix remodeling factors, and lysosomal function-associated proteins. Of note, this transcriptional signature shows limited but significant similarity to DAM in Alzheimer’s disease, indicating that different CNS diseases may share common conserved reprogramming pathways (14, 15). However, the SAE-associated state shows more prominent acute-phase response gene features and dynamic changes, reflecting the specificity of systemic inflammatory insult.

Recent progress in spatial transcriptomics technology has made it possible to link these molecular changes to particular anatomical locations. Studies have shown that brain areas most intimately linked to learning, memory, executive processing, and emotional processing, such as the hippocampal CA1 area, prefrontal cortex, and anterior cingulate cortex, show the most marked molecular reprogramming of microglia (16, 17). This spatial heterogeneity is highly associated with the loss of local synaptic markers, which provides a molecular explanation for the typical cognitive and emotional manifestations seen in SAE patients (18).

These transcriptional signatures, while sharing certain features with the DAM phenotype identified in neurodegenerative disorders, should be interpreted within a broader conceptual framework. The pathological reprogramming concept we propose extends beyond the static DAM definition in several fundamental ways. First, reprogramming emphasizes the dynamic process of transition rather than the end-state phenotype alone, encompassing how microglia arrive at the pathological state through sequential stages of triggering, signal integration, and epigenetic locking. Second, it integrates multi-level changes beyond transcription, including metabolic rewiring and functional alterations that persist even when transcriptomic profiles partially recover. Third, this framework inherently highlights reversibility and therapeutic plasticity, positioning microglia not as irreversibly damaged cells but as functionally malleable entities amenable to reeducation. This conceptual distinction has important implications for understanding microglial pathology across different neurological conditions.

### 2.2 Multi-dimensional impairment of core functions

The rearrangement of molecular identity is directly associated with the disruption of microglial physiological function. *In vivo* two-photon microscopy analysis shows that in the early phase of SAE, although the glial cell body demonstrates chemotaxis to the injury sites, the speed of movement, range of extension, and scanning rate of their complex synaptic structure are all decreased (19). This disruption of surveillance function indicates that the cells have lost the ability to perceive the microenvironment of the neural network in real time, failing to execute the early warning function and the regulation of synaptic homeostasis.

More harmful, however, is the pathological conversion of the mechanisms regulating synaptic homeostasis. Complement-mediated synaptic pruning is a vital regulatory process for neurological development and plasticity. In the inflammatory context of SAE, microglia overproduce complement components C1q, C3, and their receptors, resulting in an abnormal exaggeration of this process (20). Microglia show non-selective, excessive phagocytic activity against synaptic elements, especially the pre- and postsynaptic components of glutamatergic synapses (11). This abnormal synaptic loss is the direct pathologic basis for the reduction of synaptic density in the acute phase and for abnormalities in neural network connectivity in the chronic phase.

The clearance function of microglia shows contradictory dysregulation, as the ability to recognize and degrade particular substrates, such as apoptotic neurons and misfolded proteins, may be reduced, while the non-specific phagocytic activity may be increased inappropriately (20). This functional disorder in microglia is closely associated with the altered intracellular metabolic pathways and stress in the lysosomal system. In particular, the degree and pattern of functional disorder in microglia differ among various sepsis models, suggesting that the types and severity of infecting pathogens could affect the characteristics of this reprogramming process (21).

### 2.3 Spatio-temporal heterogeneity of pathological states

The pathological reprogramming of microglia in SAE displays complex spatiotemporal heterogeneity. From a temporal point of view, the reprogramming process displays different dynamic patterns. The acute phase is primarily characterized by a high level of inflammatory gene expression and increased phagocytic capacity (20). However, as control of the initial infection is established, certain cells can return to a homeostatic state, while other cells enter a state of reduced reactivity or senescence. The persistent functional changes in these cells can play a role in cognitive sequelae during the patient’s recovery process, related to decreased hippocampal neurogenesis and compromised synaptic plasticity (22).

**Figure 1:**
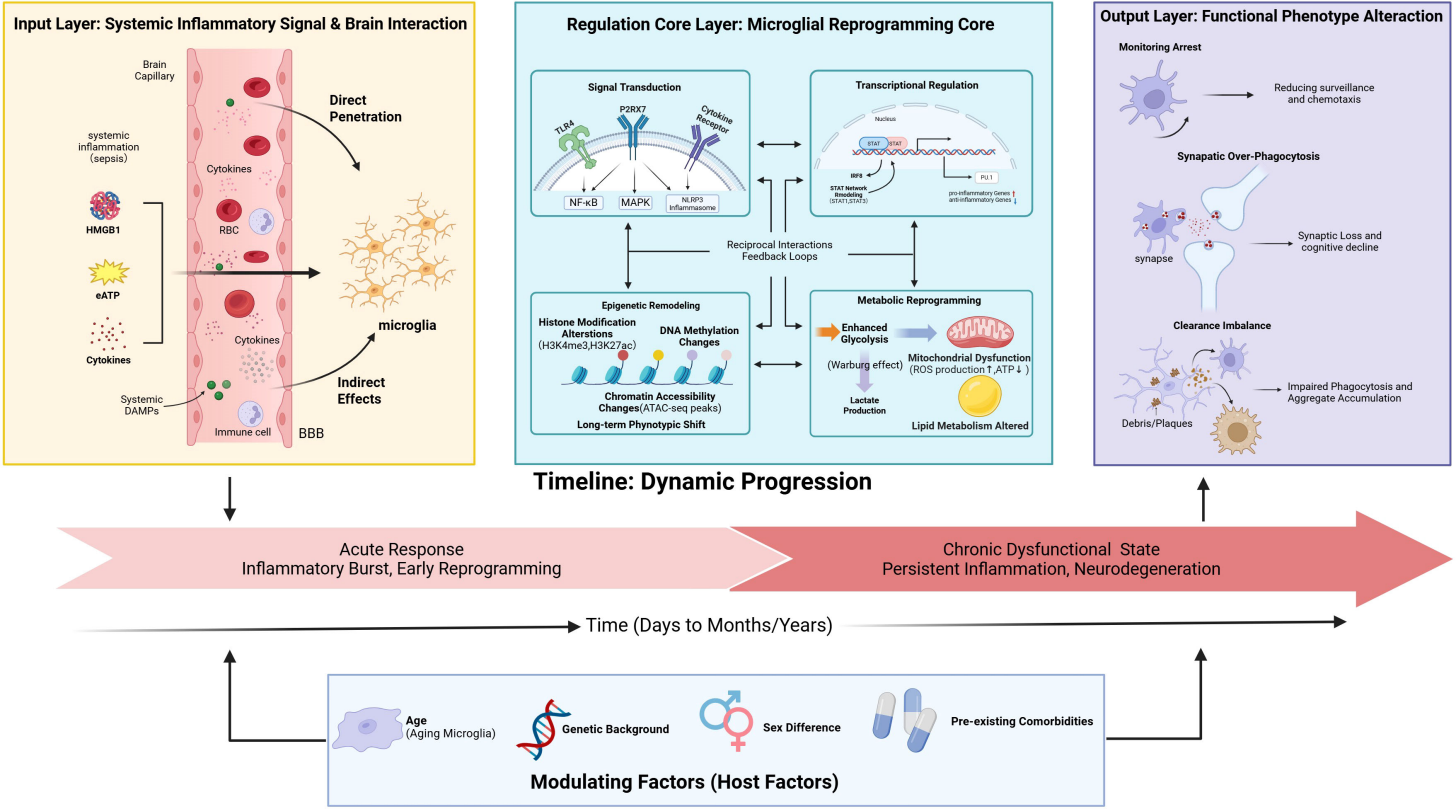
Integrated mechanisms of pathological microglial reprogramming in sepsis-associated encephalopathy. Schematic of the integrated mechanisms through which systemic inflammation induces persistent functional reprogramming of microglia. (1) Input Layer: Systemic danger signals (e.g., HMGB1, eATP, cytokines) affect microglia via circulation. (2) Regulatory Core: Illustrates the interplay of signal transduction (activation of receptors like TLR4, P2RX7), transcriptional regulation (IRF8/STAT networks), epigenetic remodeling, and metabolic reprogramming. (3) Functional Output: Reprogramming leads to impairments in core functions, including surveillance arrest, excessive synaptic phagocytosis, and clearance imbalance. (4) Timeline: Progression from acute response to chronic dysfunction. (5) Modulating Factors: Influence of host factors such as age and genetic background. SAE, sepsis-associated encephalopathy.

From a spatial perspective, the extent and pattern of microglial reprogramming exhibit pronounced heterogeneity across different brain regions. This spatial heterogeneity can be understood within the multi-layer framework presented in **Figure 1**. Regions with relatively vulnerable blood-brain barriers (BBB), such as the hippocampal CA1 region, prefrontal cortex, and cingulate cortex, show the most pronounced microglial reprogramming, suggesting that proximity to blood-borne signals is a key determinant (23). Recent studies using single-cell RNA sequencing have further confirmed that microglia in the subventricular zone and hippocampal dentate gyrus exhibit the most sensitive responses to systemic inflammation (24).

The regulation core of epigenetic and transcriptional networks may also exhibit different baseline activities across regions due to variations in local microenvironmental factors, including neuronal types and synaptic connectivity patterns. For example, glutamatergic neurons in the hippocampal CA1 region are particularly sensitive to complement-mediated synaptic pruning, consistent with the marked reduction in synaptic density observed in this region during SAE (20). Through single-nucleus transcriptomic analysis, researchers have found that neurovascular unit dysfunction in SAE patients is highly correlated with the spatial distribution of microglial reprogramming (25).

This spatial heterogeneity is particularly evident in microglial responses, where due to differences in local microenvironment, neuronal types, synaptic connectivity, and BBB properties, both the extent of microglial reprogramming and the resultant functional phenotypes vary considerably across brain regions (23). Single-cell sequencing data have also shown that within a particular brain region, different functional cell populations are present, including pro-inflammatory populations specialized for mediator production and phagocytic populations specialized for clearance (25). This functional dichotomy, if deregulated, may further affect neuroimmune homeostasis.

Host factors are increasingly being found to modulate reprogramming characteristics. Age is a significant factor that determines the pattern of microglial responses (26). In the aging brain, there is a change in the transcriptional and epigenetic status of microglia, showing increased baseline expression of inflammatory genes, which is termed the “aged” or “primed” state of microglia (27). These microglia are found to mount more severe and prolonged inflammatory responses to sepsis challenges, along with poor functional recovery (28). Genetic polymorphisms, especially those of immune receptor genes TREM2 and CD33, along with comorbid conditions of diabetes and hypertension, determine the characteristics of neuroimmune responses (29).

Synthesizing these patterns, we can delineate features that distinguish SAE-associated microglial reprogramming from general neuroinflammation. Features shared with other neuroinflammatory conditions include downregulation of homeostatic genes, upregulation of inflammatory genes, enhanced glycolysis, and altered phagocytic activity. However, SAE exhibits several distinctive characteristics: unique temporal dynamics with acute-onset persistent biphasic patterns; vascular-associated spatial distribution linked to cerebrovascular proximity (23); specific complement involvement driven by gut-derived γδT17 cell IL-17 through STING signaling (11); distinct functional consequences with preferential synaptic loss in hippocampal and prefrontal circuits; and unique molecular signatures including ADAR1 downregulation leading to aberrant RNA editing (30). These SAE-specific features suggest that therapeutic strategies may need to target pathways unique to this condition.

## 3 Regulatory mechanisms of reprogramming: integration of multilevel signaling networks

The conversion of microglia from homeostatic surveillers to pathogenic effectors in SAE is not a linear pathway but a systemic shift based on a highly interconnected signaling network. This pathological shift involves a complex process of a sequence of events: initiation by distant systemic inflammatory signals, signal transduction by specific receptors, and implementation by the drastic reorganization of intracellular transcriptional, epigenetic, and metabolic programs, culminating in a self-perpetuating dysfunctional state typified by synaptic damage (**Figure 1**). Unraveling this complex circuitry is essential for the identification of specific nodes for intervention.

### 3.1 The triggering effect of upstream danger signals

Systemic inflammation sends danger signals to the CNS through several parallel and interactive pathways. High mobility group box protein B1 (HMGB1), a major damage-associated molecular pattern molecule, is released from necrotic cells in the initial stages of sepsis (31). This initiates the activation of the nuclear factor κB (NF-κB) signal transduction pathway in microglia via Toll-like receptor 4 (TLR4). This, in turn, initiates the transcription of inflammatory genes. Adenosine triphosphate (ATP), a key extracellular signaling molecule, is released in large quantities during tissue damage and cell stress. This initiates the intracellular calcium signaling and NLRP3 inflammasome activation via the P2RX7 receptor. This leads to the release of interleukin-1β (IL-1β) and interleukin-18 (IL-18) (31, 32).

The cytokines released by peripheral immune cells affect microglial activity through both direct and indirect pathways. The cytokines present in the bloodstream, such as tumor necrosis factor-α (TNF-α) and IL-6, have a direct effect on microglia in the brain tissue by crossing the compromised BBB. However, the most significant effect of these cytokines is the enhancement of neuroinflammation by stimulating the production of chemokines and adhesion molecules by brain endothelial cells and pericytes. Recent studies have shown that the gut-brain axis plays an important role in the occurrence of SAE. Dysbiosis of the gut microbiota and its metabolic products may regulate microglial activity via the vagus nerve and humoral pathways (32, 33).

These signal molecules of danger, collectively, represent the first wave of signal molecules that trigger microglial reprogramming. Through the associated pattern recognition receptors and cytokine receptors, these signal molecules trigger the downstream transcription factors, thereby initiating the transcription of genes related to the inflammatory response, as well as the suppression of the transcription of genes related to homeostasis. It is important to note that there are substantial interactions between the different signaling pathways, thereby forming complex signaling networks.

### 3.2 Transcription and epigenetic regulation networks

The role of transcription factor networks in the regulation of microglial state transitions is significant. Interferon regulatory factor 8 (IRF8), a significant transcription factor in the regulation of microglial lineage commitment and functional maintenance, shows altered expression levels and activity in SAE, thereby affecting the expression of target genes (34). Signal transducer and activator of transcription (STAT) family members, such as STAT1 and STAT3, have significant functions in cytokine signaling and the expression of inflammatory genes (35). Recently, the function of nuclear receptor family members, such as the liver X receptor (LXR) and peroxisome proliferator-activated receptor gamma (PPARγ), in the regulation of microglial lipid metabolism and inflammatory responses was identified, and alterations in their activity could affect reprogramming pathways (36).

Epigenetic regulatory mechanisms have been demonstrated to play a pivotal role in the maintenance of pathological states. Chromatin immunoprecipitation sequencing analysis revealed global alterations in histone modification patterns within microglia during SAE. Increased H3K27 acetylation has been observed to correlate with open promoter states of inflammatory genes, while H3K9 trimethylation has been implicated as a contributing factor to the silencing of homeostatic genes (37, 38). These epigenetic modifications provide a mechanistic basis for the altered gene expression patterns observed in SAE microglia, and as discussed below, can persist long after the initial insult.

Recent studies have begun to elucidate the specific epigenetic programs underlying different forms of microglial memory. The concepts of microglial priming and tolerance, collectively termed innate immune memory, have been extensively reviewed in the literature (39–41). Zhang et al. systematically mapped the epigenetic landscape of microglial innate immune memory, demonstrating that LPS preconditioning-induced tolerance is associated with sustained reductions in H3K4me3 and H3K27ac at inflammatory gene loci, accompanied by decreased chromatin accessibility that persists for weeks (42). Schaafsma et al. further identified that RelB-dependent epigenetic silencing mechanisms mediate long-lasting anti-inflammatory suppression following LPS preconditioning (43). These findings reveal that microglial responses to inflammatory stimuli are not simply on-off switches but are encoded in durable epigenetic modifications that shape future responses, a concept with direct relevance to SAE where the initial septic insult may leave an epigenetic “scar” that programs subsequent microglial behavior.

Dynamic changes in DNA methylation play a part in the regulation of microglial reprogramming. DNA methylation was analyzed on a genome-wide scale to examine DNA methylation status changes induced by sepsis. The results showed that sepsis induced changes in methylation status at thousands of CpG sites in microglia. The changes occurred in gene regions related to immune responses, cell metabolism, and synaptic function. The demethylation of the promoter regions of some inflammation-related genes may reduce their thresholds of transcriptional activation, leading to hyperreactivity of cells to stimulation (44).

### 3.3 Rewiring metabolic pathways for functional support

Metabolic reprogramming furnishes the necessary energy and biosynthetic resources to execute the pathological functions of microglia (45). In SAE, the metabolic pathway of glial cells changes from oxidative phosphorylation to glycolysis, a phenomenon similar to the Warburg effect. This increased glycolysis furnishes rapid energy in the form of ATP, which is used to execute the energetic functions of the cell, such as phagocytosis, migration, and synthesis of inflammatory mediators (46, 47). In addition, elevated glycolytic intermediate levels, including those used in the pentose phosphate pathway, serve as precursors for nucleotide synthesis and antioxidant glutathione synthesis (48).

The dynamics and function of mitochondria also undergo significant changes during this metabolic reprogramming. In microglia, mitochondria exhibit significant tendencies for fragmentation, depolarization of mitochondrial membranes, and increased production of reactive oxygen species (ROS) (49). These changes not only affect mitochondrial function but also play a significant role in regulating inflammasome activation and cell fate decisions. Importantly, certain metabolic intermediates such as succinate and itaconic acid can act as signaling molecules to regulate inflammation, thereby establishing a feedback loop between metabolism and inflammation (50).

Additionally, the abnormal metabolism of lipids should also be taken into consideration. The glial cells of SAE display abnormal cholesterol metabolism and the accumulation of lipid droplets, which could compromise the fluidity of cell membranes and the efficiency of signal transduction (49). Recent studies showed that the expression of some enzymes involved in lipid metabolism, such as cholesterol 25-hydroxylase, might be involved in the regulation of glial cell inflammatory responses by producing oxidized sterol molecules via autocrine or paracrine mechanisms (51).

### 3.4 Modulation and amplification effects in the aging microenvironment

Aging fundamentally alters microglial function, thereby significantly influencing their response to sepsis. The aging microglia have characteristic changes in the transcriptome and epigenome, showing increased baseline expression of inflammatory genes, decreased phagocytic and migrating ability, and mitochondrial dysfunction. When these already aged microglia are subjected to the strong challenge of sepsis, it is likely that their inflammatory responses are exacerbated (52).

The issue of cellular senescence in SAE has slowly attracted attention. Karabag et al., using senescence-associated β-galactosidase staining and p16INK4a expression analysis, demonstrated that sepsis can induce a subset of microglia to enter a senescent state. These senescent microglia release inflammatory cytokines, chemokines, and proteases through the senescence-associated secretory phenotype (SASP), altering the local microenvironment and affecting neuronal survival (53). This effect of microglia on neurons could cause further neuroinflammation, leading to neuronal injury and death (54, 55). The relationship between cellular senescence and acute inflammation is likely a major cause of the high incidence of SAE in the elderly population (54).

## 4 Treatment strategy

**Figure 2:**
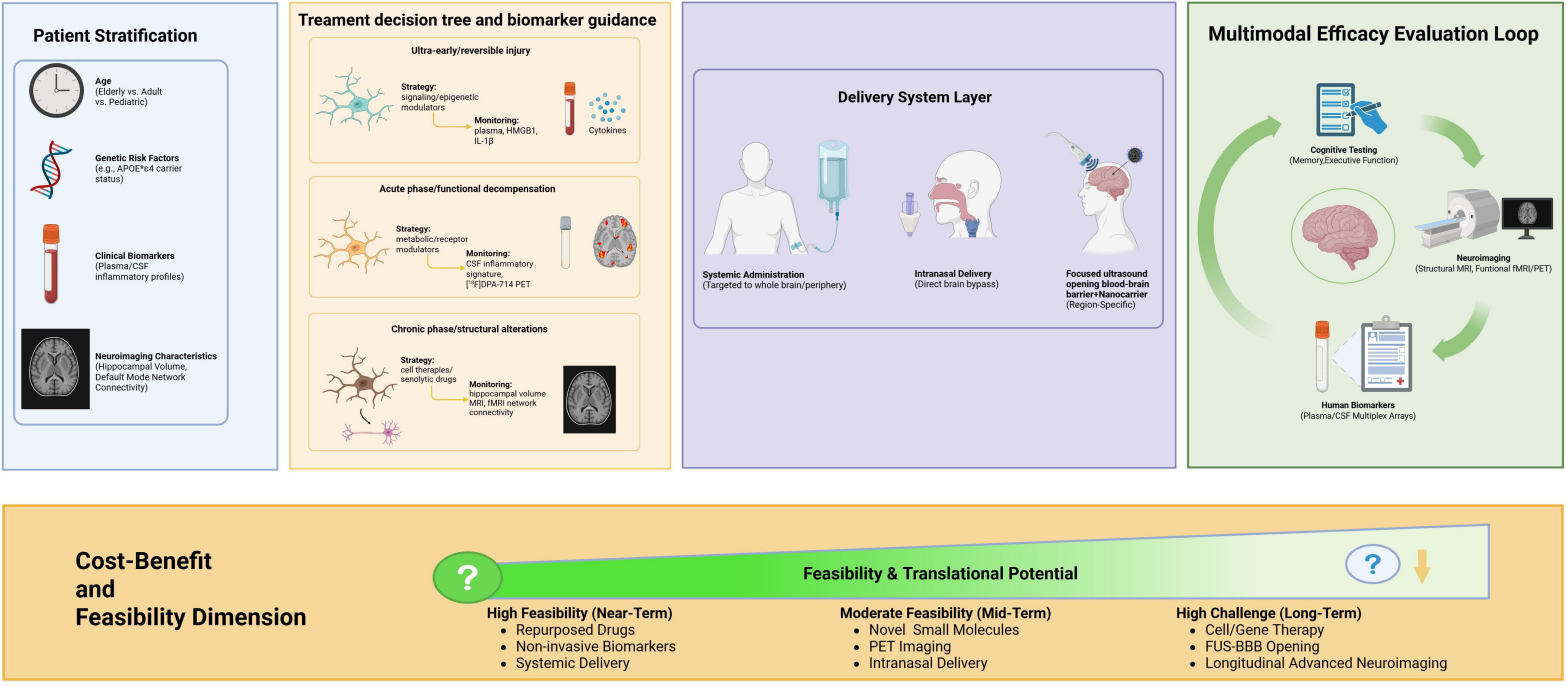
Translational roadmap for targeting microglial reprogramming in sepsis-associated encephalopathy therapy. Overview of the translational pathway from basic research to clinical application. (1) Patient Stratification: Subgrouping patients based on biomarkers (e.g., genetic risk, inflammatory profiles, neuroimaging). (2) Therapeutic Decision Tree: Selection of intervention strategies targeting signaling, epigenetics, metabolism, or cells according to disease stage (hyper-acute, acute, chronic) and biomarkers. (3) Delivery Systems: Methods to overcome the blood-brain barrier. (4) Efficacy Evaluation Loop: Dynamic treatment adjustment via multimodal assessment. (5) Translational Potential: Color gradient indicates the near-term translatability of different strategies.

However, to transition from mechanistic insight to clinical application, there is a need for a strategic approach that takes into account the dynamic nature of SAE and the variability of patient responses. In this regard, we would like to present a roadmap for precision medicine that provides a logical sequence from patient stratification based on biomarkers, through stage-adaptive selection of therapeutic approaches aimed at different nodes of the reprogramming circuitry, to closed-loop evaluation of efficacy (**Figure 2**). The subsequent sections will describe the interventions corresponding to this translational strategy.

### 4.1 Pharmacological intervention: reversing pathological processes

Pharmacological interventions targeting microglial reprogramming primarily modulate epigenetic and metabolic pathways. For instance, histone deacetylase inhibitors such as vorinostat and romidepsin increase chromatin acetylation levels, thereby reversing inflammatory gene expression and restoring homeostatic gene expression (56, 57). Preclinical studies have demonstrated that these pharmacological agents improve microglial functional status in animal models of sepsis, reducing synaptic loss and cognitive impairments. In addition, bromodomain and extra-terminal domain protein inhibitors such as JQ1 have been shown to have better specificity in microglia by selectively inhibiting inflammatory gene transcription by interfering with transcription coactivator recruitment (58).

Another potential pharmacological strategy is metabolic modulation. Adenosine monophosphate-activated protein kinase agonists, such as metformin, have been shown to restore cellular energy homeostasis and alleviate inflammation (59). The effect of mammalian target of rapamycin (mTOR) inhibitors, such as rapamycin, on microglia functional phenotypes is related to the regulation of cell growth and autophagy (60, 61). It should be noted that the effects of these metabolic regulators often depend on certain contexts, and their optimal timing and dosage in SAE treatment need to be studied.

Targeting a specific receptor has shown promise in terms of translational potential. The agonists of triggering receptors expressed on myeloid cells 2 (TREM2), such as the monoclonal antibody AL002c, have been shown to enhance microglia-mediated beneficial phagocytic activities, thereby removing pathological proteins such as Aβ (62, 63). The proven safety of such compounds in Alzheimer’s disease clinical trials is also valuable for SAE treatment (64). Antagonists of the purinergic receptor, such as Brilliant Blue G, have been shown to inhibit excessive activation of ATP receptors, which leads to reduced inflammatory reactions (65). These compounds represent a paradigm shift in neuroimmunotherapy from immunosuppression to immune reprogramming by modulating the functional status of microglia, rather than suppressing them. The translational challenges associated with these approaches, including BBB penetration and optimal treatment timing, will be discussed in Section 4.4.

### 4.2 Genetic engineering and synthetic biology strategies

Recent advances in gene editing technologies have offered unparalleled opportunities to control microglial functions with high precision. In addition, functional genomics screening using the clustered regularly interspaced short palindromic repeats (CRISPR) system and its associated proteins have been used to identify multiple crucial genes and pathways involved in microglial reprogramming. In this regard, recent studies have identified the critical role of the epigenetic regulator TET2 in microglial inflammatory responses, and its loss is associated with the progression of neurodegenerative diseases (66).

Synthetic biology strategies can also facilitate the development of more complex and intelligent therapeutic interventions. For example, gene expression systems can be designed in microglia that respond to inflammatory signals by expressing anti-inflammatory cytokines, such as IL-10 or transforming growth factor-β (TGF-β) (67). More complex synthetic biology strategies can also be used to develop gene expression systems that respond to specific combinations of environmental signals, enabling microglia to make more precise responses to complex pathological cues. For example, therapeutic gene expression can be designed to occur in microglia preferentially under a pro-inflammatory milieu characterized by high levels of TNF-α and low levels of interferon-γ (IFN-γ) (68).

Chimeric antigen receptor engineered microglia represent an advanced approach in cell therapy. In brain tumor therapy, macrophages expressing epidermal growth factor receptor variant III specific chimeric antigen receptors have shown promising antitumor effects by enabling targeted recognition and elimination of tumor cells (69, 70). This paradigm of receptor-directed targeting can be conceptually extended to SAE. Instead of targeting tumor antigens, chimeric antigen receptors could be engineered to recognize specific damage-associated molecular patterns or pro-inflammatory mediators that drive SAE pathogenesis. For example, chimeric antigen receptor microglia expressing receptors against HMGB1 or specific complement components such as C1q and C3 could selectively neutralize these pathogenic molecules at the site of neuroinflammation, while preserving the physiological functions of these mediators in other contexts (31, 32). This approach would transform microglia from non-specific responders that broadly engulf synaptic elements into precisely targeted therapeutic effectors that specifically clear pathological molecules. Although this remains a conceptual framework in SAE, it illustrates how synthetic biology strategies could achieve the goal of precision immunomodulation, targeting pathogenic factors while sparing physiological functions.

### 4.3 Cell replacement and regenerative medicine strategies

The basic rationale behind the application of cell replacement therapy is its potential to modify the immunological microenvironment of the CNS by replacing dysfunctional microglial cells. Colony-stimulating factor-1 receptor antagonists such as PLX5622 are highly efficient in the selective and reversible removal of the majority of the endogenous microglial population, which makes way for the transplantation of exogenous cells (71). It has been shown that the transplantation of healthy progenitor cells following the removal of endogenous microglial cells improves the phenotypes of various neurological disease models (72). However, the efficacy of this approach depends on the survival of the transplanted cells.

The mechanisms underlying microglial repopulation after acute colony-stimulating factor 1(CSF1) receptor inhibitor treatment have been extensively studied. Research has demonstrated that microglial repopulation in the brain following PLX3397 treatment derives entirely from proliferation of residual microglia, with no contribution from bone marrow derived cells or border associated macrophages (73). Subsequent studies using parabiosis models and fate mapping techniques further confirmed that repopulated microglia after acute depletion originate solely from self-renewal of residual parenchymal cells (74). Novel genetic tool mice have reached the same conclusion (75). This finding carries dual implications. On the positive side, it indicates that the microglial pool is closed under steady-state conditions, preventing peripheral cells from contaminating the brain parenchyma and preserving the uniqueness of the CNS immune microenvironment. From a therapeutic perspective, this constitutes a major challenge. If the goal is to introduce genetically modified exogenous cells or healthy donor cells, CSF1 receptor inhibitor treatment alone is insufficient. Residual endogenous microglia rapidly proliferate to fill the niche, preventing transplanted cell engraftment.

Achieving engraftment of peripherally derived cells in the brain requires more intensive preconditioning regimens. Studies have found that chronic, long term CSF1 receptor inhibitor treatment, in the absence of irradiation, can induce increased BBB permeability, allowing limited entry of peripheral macrophages into the brain parenchyma (76). Comparisons of different preconditioning protocols for peripheral cell engraftment have revealed that irradiation combined with CSF1 receptor inhibitors achieves the highest level of peripheral cell replacement (77). Recent reports indicate that combined irradiation and CSF1 receptor inhibitor treatment enables brain wide engraftment of hematopoietic stem cell derived microglia like cells, successfully correcting phenotypes in progranulin deficient mice (78). However, these intensive preconditioning protocols themselves carry significant pathophysiological consequences. Studies have reported that CSF1 receptor inhibitor combined with irradiation induces irreversible neuroglial remodeling in the retina (79). In the absence of TGFβ signaling, engraftment of peripherally derived macrophages can lead to fatal demyelinating disease (80). These findings caution that cell replacement strategies must strike a delicate balance between efficacy and safety.

A more thought-provoking recent discovery is that even after microglial depletion and repopulation, certain pathological phenotypes or immune memories may persist. Recent studies have demonstrated that microglia rendered endotoxin tolerant by LPS exposure retained their tolerant phenotype even after forced depletion and repopulation using CSF1 receptor inhibitors (81). Repopulated microglia still exhibited attenuated inflammatory responses to subsequent LPS challenge. This suggests that innate immune memory may be transmitted through epigenetic mechanisms in the few residual microglia and reestablished in the repopulated population. Investigations of forced turnover of aged microglia have found that although cells were replaced by fresh, young derived microglia, certain signals in the brain tissue microenvironment such as CD22 and senescence associated secretory phenotype factors persisted, resulting in repopulated microglia still exhibiting partial aged phenotypes (82). This indicates that cell replacement therapy requires not only replacing the cells themselves but also correcting the microenvironmental signals that maintain pathological states.

The improvement of induced pluripotent stem cell technology has helped to produce large numbers of human-derived microglia. By optimizing the differentiation conditions, it is possible to obtain microglia from induced pluripotent stem cells that are similar to primary microglia at the transcriptional and functional levels. The cells can be genetically engineered prior to transplantation to improve their neuroprotective capabilities (83). This can be achieved by overexpressing brain-derived neurotrophic factor (BDNF) or glial cell-derived neurotrophic factor (GDNF) so that the transplanted cells are capable of providing neurotrophic support, apart from replacing the dysfunctional endogenous cells (84).

A more advanced technique is the direct reprogramming *in vivo*. By using specific combinations of transcription factors, microglia can be reprogrammed from a pathological state to a neuroprotective state without the need to go through the complicated procedures of removing the cells and transplanting them (71). Although still at the initial stages, the ultimate goal of microglia regulation is precisely the specific regulation of the cells at the right time, the right place, and the right manner.

### 4.4 Transformation challenges and comprehensive strategies

However, the existing treatment approaches are challenged in their translation to the clinic. **Table 1** gives a comparative insight into these approaches, integrating their fundamental mechanisms, pivotal evidence, development stage in SAE research, and limitations. This landscape analysis identifies several interlinked hurdles. The BBB is the foremost barrier, impeding the passage of most biologics and cellular products, requiring the development of targeted delivery methods using focused ultrasound-mediated opening or functionalized nanoparticles. Equally important is the lack of definition of the therapeutic window, where treatments working in the hyperinflammatory phase become ineffective or even toxic in the later stages, requiring the development of dynamic biomarkers to time these interventions. Moreover, the mismatch between young and healthy animal models and the elderly and comorbid human SAE population makes preclinical studies less informative.

**Table 1:** Therapeutic strategies targeting microglial inflammatory reprogramming in SAE.

Strategy category	Representative approach	Core mechanism	Research status (in SAE)	Key considerations	Reference
Pharmacological Modulation	HDAC inhibitor (Vorinostat/SAHA)	Epigenetic regulation (histone acetylation); reversal of inflammatory transcription	Preliminary validation in animal models, showing attenuated cognitive dysfunction and microglial activation induced by peripheral inflammation. Effects are dose and context-dependent.	BBB penetration; treatment timing; specificity of action	(56, 57)
	TREM2 agonist (AL002c)	Activation of TREM2-DAP12 pathway; enhancement of protective phagocytosis	Primarily mechanistic exploration in neurodegenerative models (e.g., Alzheimer’s). Direct evidence in SAE remains limited but is a putative target.	Specificity to SAE pathology; unknown safety profile	(62, 64)
Genetic & Cellular Engineering	CRISPR-based functional screen	Systematic identification of key regulatory genes	Used for target discovery in microglia biology *in vitro*. Application in SAE models is an emerging frontier.	*In vivo* delivery challenges; editing safety	(66)
	AAV-mediated gene therapy (e.g., IL-10)	Sustained anti-inflammatory cytokine expression in glia	Proof-of-concept established in other neuroinflammatory diseases. Not yet directly tested in SAE models.	Need for whole-brain delivery; immunogenicity risk	(67, 85)
	iPSC-derived microglia transplantation	Cell replacement and functional supplementation	Early exploration in chronic disease models. Direct application in acute SAE is highly experimental.	Graft survival and integration; tumorigenic risk; high cost	(83, 84)
Endogenous Modulation	G-CSF mobilization	Mobilization of bone marrow progenitors; indirect modulation of brain milieu	Conflicting evidence from animal models, with mixed results on outcomes. Its role in SAE is not well-defined.	Extremely low brain entry efficiency; may mobilize pro-inflammatory cells	(86)

SAE, Sepsis-associated encephalopathy; HDAC, Histone deacetylase; LPS, Lipopolysaccharide; AAV, Adeno-associated virus; iPSC, Induced pluripotent stem cell; G-CSF, Granulocyte colony-stimulating factor; BBB, Blood-brain barrier.

The complexity of SAE pathophysiology implies that successful treatment is likely to involve a rational combination or sequential approaches. For example, a treatment course might consist of an acute phase signal inhib itor to counteract the initial insult, followed by an epigenetic modulator to correct pathological programming, and finally a pro-repair agent to facilitate recovery (87, 88). The success of this strategy not only depends on the discovery of synergistic combinations but also on the need for personalized therapy. Future therapies must take into consideration patient-specific variables such as age, genetic makeup, and comorbidities through biomarker-based stratification and delivery systems capable of adapting to the dynamic brain microenvironment in sepsis (89).

## 5 Conclusion and outlook

Pathological reprogramming of microglia in SAE is a complex, dynamic process that occurs at multiple levels, from systemic changes in molecular expression to cellular function. This theoretical approach not only helps us better understand the underlying mechanisms of the disease but also redefines the role of microglia from passive inflammatory participants to dynamic therapeutic targets (9). This framework provides a foundation for developing new therapeutic strategies, including reprogramming the functional status of these cells and restoring their neuroprotective properties. The paradigm shift from pathological reprogramming to therapeutic reprogramming reflects the evolution of neuroimmunotherapy from traditional immunosuppression to more accurate immune remodeling.

Nevertheless, this pathway also encounters a number of challenges. For instance, there is a wide gap between model systems and clinical settings, where most SAE mechanisms and therapy explorations have used young and healthy rodents, while SAE patients in clinical settings are mostly elderly individuals suffering from a number of comorbid conditions. For instance, the relationship between aging-associated glial cell senescence and sepsis-induced reprogramming remains a “weak link” in current research (11). On the other hand, the precise timing of interventions also remains a challenge, as the extent of the reversibility of the reprogramming process and the existence of “decisive” epigenetic and metabolic “tipping points” directly affect the choice of intervention time windows. Technical barriers also remain a significant challenge, as the BBB remains a barrier for the effective administration of most macromolecular drugs, gene editing tools, and cell therapy approaches (90). For instance, while emerging technologies such as “focused ultrasound” and “nanocarriers” hold promise, their safety, efficacy, and brain region specificity require further validation (90). In addition, the issue of personalized treatment also remains a challenge, as microglial responses in SAE patients depend on a number of factors, including age, genetic background, comorbid conditions, and types of pathogens (91).

Looking forward, it is imperative that several major avenues of research are advanced with urgency. The first is to gather human data by undertaking spatial multi-omics studies on post-mortem brain tissue from SAE patients to directly validate the reprogramming characteristics that have been observed in animal models (92). The second is to utilize humanized mouse models and brain organoids to more closely mimic human responses. The third major avenue is to develop dynamic monitoring tools. This includes the development of new PET tracers, exosomal markers for microglia in cerebrospinal fluid or blood, and functional network markers using EEG or fMRI to monitor the status of microglia and treatment response in real time (11). Due to the complexity of SAE, it is possible that a single approach may not be enough. Future studies may involve a combination or sequential use of a variety of strategies, such as correcting endogenous cell function and then providing long-term protection via genetically engineered cells, or removing senescent cells and then replacing them with healthy ones. Finally, there will be a convergence of breakthroughs from various disciplines into the field of SAE therapy research, combining recent advances in systems biology, synthetic immunology, and biomaterials science. These include the design of smart nanoparticles that can sense the local inflammatory environment to release drugs or the development of engineered microglia with multi-sensory and multi-response functions (90).

In summary, SAE represents a mechanistically clear and time-defined *in vivo* model for studying the reprogramming of tissue-resident immune cells in the context of inflammation. The reprogramming of microglia in this context can be seen as the ultimate cellular manifestation of the shift from acute defense to chronic dysfunction in the inflammatory response. Thus, investigating the mechanisms and therapeutic strategies underlying microglial reprogramming in SAE extends beyond SAE itself—offering not only conceptual frameworks but also experimentally tractable approaches to deciphering adaptive and maladaptive immune responses in other tissues during systemic inflammation, and to elucidating how aging and metabolic dysregulation modulate these responses.

However, it is important to maintain a critical perspective and remember that, although this reprogramming framework has tremendous implications, microglia are just one component of the neuroimmune axis and their multifaceted interactions with astrocytes, other peripheral immune cells, neurons, and the BBB are also essential for SAE pathogenesis. This emphasis on the re-education of microglia might also lead to other important pathological processes being ignored. This approach to microglia as a “programmable therapeutic platform” has implications that stretch beyond SAE and could have important cross-disciplinary implications for other neurological diseases, such as Alzheimer’s and traumatic brain injury. Further research into their reprogramming processes and the development of precision control strategies could lead to the intelligent management of the immune system within the CNS and represents not only a key to improving patient outcomes for SAE patients but also a crucial step towards healthy longevity and aging. Realizing this potential will require coordinated progress across three interdependent domains: mechanistic understanding, therapeutic innovation, and rigorous preclinical and clinical validation—ultimately bridging the gap between theoretical reprogramming paradigms and tangible, patient-centered impact.
